# Cost–effectiveness of apixaban and warfarin in the prevention of thromboembolic complications among atrial fibrillation patients

**DOI:** 10.1186/s40064-016-3024-5

**Published:** 2016-08-17

**Authors:** Taru Hallinen, Erkki J. Soini, Miika Linna, Samuli I. Saarni

**Affiliations:** 1 ESiOR Oy, Tulliportinkatu 2 LT4, 70100 Kuopio, Finland; 2 HEMA/Aalto University, TUAS-talo, Otaniementie 17, Espoo, Finland; 3 Turku University Hospital and University of Turku, P.O. Box 52, 20521 Turku, Finland

**Keywords:** Anticoagulation, Apixaban, Atrial fibrillation, Cost–utility, Stroke, Warfarin

## Abstract

**Background:**

To reduce the risk of thromboembolic complications, clinical guidelines recommend anticoagulation treatment for almost all atrial fibrillation (AF) patients. Although warfarin has long been the primary treatment alternative, now newer alternatives such as apixaban have proven effective in prevention of the thromboembolic complications of non-valvular AF. The aim of this study is to assess the cost–effectiveness of apixaban when compared with warfarin in the prevention of AF-associated thromboembolic complications in Finland.

**Methods:**

The assessment was performed with a lifetime Markov-model with the following health states: non-valvular AF, ischemic stroke, hemorrhagic stroke, other intracranial bleed, other major bleed, clinically relevant non-major bleed, myocardial infarction, and systemic embolism. The treatment efficacies were obtained from the ARISTOTLE trial. Representative Finnish input data were used for the model states, including background mortality, resource use, costs (in 2014 values), and EQ-5D-3L-based quality of life. The results (with 3 % annual discounting) are presented as incremental cost–effectiveness ratios [ICER, cost per quality-adjusted life year (QALY) gained], the expected value of perfect information (EVPI), and the probability of apixaban being cost–effective at various willingness-to-pay levels.

**Results:**

Apixaban increased life-expectancy by 0.17 years and quality-adjusted life-expectancy by 0.14 QALYs when compared with warfarin. Additional QALY was gained with apixaban at a cost of 1824 euros based on the deterministic analysis. The maximum EVPI was 649 euros/patient at 1282 euros per QALY gained in the probabilistic analysis. The probability of apixaban being cost–effective reached 80 % when the willingness-to-pay per QALY gained was 14,857 euros. In deterministic sensitivity analyses, ICERs varied from dominance of apixaban to additional QALY being gained at a cost of 12,312 euros.

**Conclusions:**

The ICERs obtained were well below the WHO-CHOICE threshold values for cost–effective interventions, suggesting that apixaban is a very cost–effective treatment alternative for warfarin in Finnish patients with AF.

## Background

Atrial fibrillation (AF) is a major risk factor for stroke, increasing the risk of stroke approximately fivefold (Wolf et al. 1991). In Finland, approximately 13 % of all patients with ischemic stroke have a prior diagnosis of AF (Meretoja et al. 2011). Warfarin has long been the recommended antithrombotic treatment for AF patients as it reduces the risk of stroke by approximately 60 % (Hart et al. 2007). However, characteristics of warfarin including frequent monitoring of international normalized ratio (INR) and numerous drug interactions limit its use. Recent Finnish studies suggest that warfarin is underused among AF patients. Approximately 50 % of all AF patients for whom anticoagulation is recommended in the Finnish Current Care Guideline (2015) based on their stroke risk (i.e. CHA_2_DS_2_-VASc ≥ 1, see e.g. Lip et al. 2010) were treated with warfarin in one municipality (Hallinen et al. 2014) and 30 % of the previously diagnosed AF patients admitted to Finnish emergency departments due to AF did not receive warfarin treatment at the time of hospitalization despite their moderate to high risk of stroke (Lehto et al. 2011).

New anticoagulant treatments such as apixaban, dabigatran, and rivaroxaban have all proven effective in the prevention of thromboembolic complications in patients with non-valvular AF (Granger et al. 2011; Connolly et al. 2009; Patel et al. 2011). In Finland and many other countries the decision on whether to publicly fund the use of these drugs is at least partially based on the cost–effectiveness of the drug against the most-used treatment. In Finland, the costs of these new anticoagulants are currently reimbursed (35 % of costs are covered by the Social Insurance Institution) for patients with high risk of embolism (i.e. CHA_2_DS_2_-VASc ≥ 2) and patients with moderate risk of embolism (i.e. CHA_2_DS_2_-VASc = 1) when warfarin cannot be used due to its side-effects or interactions or when patients’ INR-values during stabilized warfarin treatment remain in the target range less than 70 % of the time (Kela 2015).

The aim of this study was to assess the cost–effectiveness of apixaban when compared with warfarin in the prevention of thromboembolic complications in Finnish AF patients. The study applies previously unpublished health care costs and quality of life estimates that have been observed for Finnish patients in real-life setting.

## Results

**Fig. 1:**
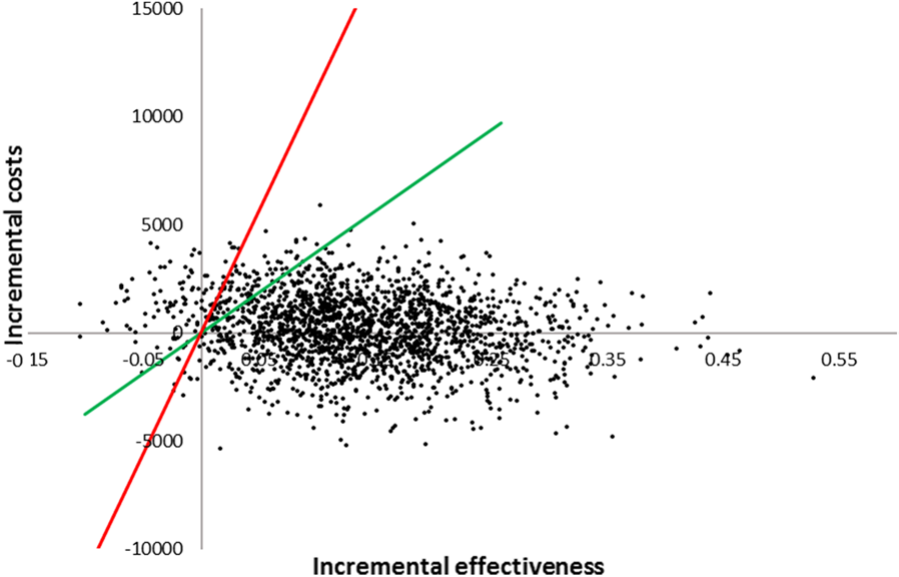
The cost-effectiveness plane for apixaban versus warfarin. Green line depicts ICER threshold equal to the Finnish GDP and red line depicts ICER threshold equal to 3 times the Finnish GDP

Apixaban use increased life-expectancy and quality adjusted life-expectancy on average by 0.17 and 0.14 years, respectively, when compared with warfarin (see Table 1). These gains were reached at an additional cost of 261 euros during patient’s life-time. An additional quality-adjusted life year (QALY) was therefore gained at a cost of 1824 euros, which is clearly below the commonly used threshold values for incremental cost–effectiveness ratios (ICERs) that are considered to support claims of cost–effectiveness. The cost–effectiveness plane illustrating differences in costs and effects between apixaban and warfarin is shown in Fig. 1.

**Table 1:** Results of the cost–effectiveness analyses

	Lifetime costs (€)			Lifetime QALYs			ICER (€/QALY gained)
	Apixaban	Warfarin	Diff.	Apixaban	Warfarin	Diff.	
Base case	16,197	15,936	261	7.19	7.05	0.14	1824
Without discounting	20,647	20,442	205	8.99	8.80	0.19	1060
*Sensitivity analyses*							
CHADS 0–1	15,491	14,931	560	7.25	7.12	0.13	4347
CHADS 2	16,151	15,708	443	7.20	7.07	0.13	3387
CHADS 3–6	17,012	17,266	−254	7.13	6.96	0.17	Dominant
TTR < 52.38 %	16,047	17,150	−1104	7.19	6.97	0.22	Dominant
52.38 % ≤ TTR < 66.02 %	17,397	16,170	1226	7.14	7.04	0.10	12,312
66.02 % ≤ TTR < 76.51 %	16,164	15,131	1033	7.20	7.10	0.10	10,386
TTR ≥ 76.51 %	15,150	15,249	−98.23	7.25	7.10	0.15	Dominant
No treatment discontinuations after trial period	19,473	19,042	431	7.24	7.04	0.21	2102
Warfarin monitoring cost −50 %	16,197	14,650	1546	7.19	7.05	0.15	10,817
Warfarin monitoring cost +50 %	16,197	17,222	−1025	7.19	7.05	0.14	Dominant

**Fig. 2:**
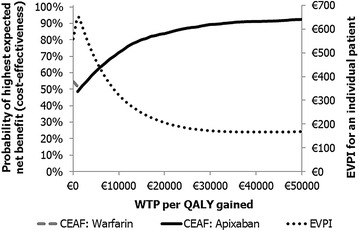
Cost-effectiveness acceptability frontier (CEAF) and expected value of information (EVPI) curves for the analysis. WTP=willingness to pay

The cost–effectiveness acceptability frontier (CEAF) in Fig. 2 shows the probability of the optimal treatment being the most cost–effective treatment alternative at various willingness to pay (WTP) thresholds per QALY gained. The probability of apixaban being cost–effective reached 80 % when the WTP per QALY gained was 14,857 euros. Apixaban had 91 and 94 % probability for cost–effectiveness when the WTP was 37,576 and 112,728 euros per QALY gained, respectively. Based on the probabilistic simulation results and Bayesian treatment ranking, apixaban had 95 % probability of being the first best option in terms of highest QALYs and had 45 % probability for the lowest costs.

As can be seen from Fig. 2, expected value of perfect information (EVPI) reached its maximum value of 649 (2.5–97.5 % percentile 0–3409) euros per patient when the probabilistic ICER was 1282 euros per QALY gained. The EVPI was 166 (0–2556) and 234 (0–3474) euros per patient when WTP was 37,576 and 112,728 euros per QALY gained, respectively.

In deterministic sensitivity analyses ICERs varied between negative values, i.e. apixaban dominated warfarin, and 12,312 euros per QALY gained. The highest ICERs were obtained in scenarios where apixaban was compared with warfarin treatment with intermediate percentage times spent in the therapeutic INR range (TTRs) and when the cost of monitoring for warfarin was reduced by 50 %. Even the highest ICERs obtained were well below the common official international and unofficial Finnish cost–effectiveness thresholds of 30,000 and 47,000 euros per QALY gained.

## Discussion

Apixaban use increased average treatment costs, life-expectancy, and quality-adjusted life-expectancy of AF patients when compared with warfarin. An additional QALY was gained with apixaban at a cost of 1824 euros. Based on WHO-CHOICE threshold values for cost–effectiveness (WHO 2016), our analysis suggests that apixaban can be considered a very cost–effective treatment alternative to warfarin in Finland. Based on a recent systematic literature review of cost–effectiveness studies of the new oral anticoagulants, our finding is in line with published assessments from other countries (Kansal et al. 2013).

Based on the extensive sensitivity analyses and CEAF, the results obtained were relatively robust and the probability of apixaban being cost–effective was high. The expected value of obtaining additional information for uncertain probabilistic model parameters remained relatively low and was around 649 euros per patient at maximum. This finding suggests that further research to reduce the uncertainty would be worthwhile only if the costs associated with further research were less than 649 euros per study participant.

Perhaps the most interesting practical finding of our study was the non-linearity of ICERs with regard to warfarin treatment quality. ICERs were lowest when the TTR was at either the higher or lower end of the spectrum. The highest ICERs were observed for intermediate TTRs. Although the subgroups analyses of the ARISTOTLE trial were not powered to detect significant differences between warfarin and apixaban in these TTR subgroups, it seems possible that in centers with high TTR values the patients may have been at higher risk of bleeds when compared with centers with lower TTRs. Because apixaban use is associated with a lower risk of bleeds when compared with warfarin, this observation could explain the improvement in the cost–effectiveness of apixaban as TTR with warfarin increases. Interestingly, a recent Finnish study (Soini et al. 2013) observed a potential association between higher TTR and lower quality of life (QoL), perhaps reflecting a similar phenomenon.

On a broader perspective our assessment shows that QALYs gained during anticoagulation treatment are associated with reasonable costs in relation to benefits gained regardless of whether the patients are treated with apixaban or warfarin. In fact, our analysis illustrates a rare case (see Soini et al. 2012) in which the ICER between compared treatments is very similar to the average cost–effectiveness ratios (CER, i.e. cost/QALY for each comparator) for apixaban (2253 euros) and warfarin (2260 euros). Based on the Bayesian treatment ranking of lowest CER, the probability of being the first best option was 53 % for apixaban and 47 % for warfarin. Even though the CERs implicitly reflect a comparison setting in which the comparator to given treatment would be “instant death” without any costs or QALYs (Soini et al. 2012), very high CERs may indicate that the treatment would not be considered acceptable from cost perspective (an exception to this interpretation would be the case in which the choice to “do nothing” or sc. “instant death” would lead to higher CER). In our study, this is clearly not an issue for either treatment. In the absence of common comparator, relative benefit of apixaban and warfarin versus the common comparator (e.g. placebo) could not be estimated and, thus, relative benefit–CER (see Soini et al. 2016) or ICERs in terms of given treatment versus the common comparator were not implemented in this study. In fact, because warfarin was the recommended treatment practice at the time of ARISTOTLE trial, a placebo-controlled trial would have been unethical.

Our study has some important strengths. We collected both the cost and QoL data from representative Finnish data sources, thereby increasing the validity of our results in the Finnish context. Furthermore, the patient characteristics and risk factors in the ARISTOTLE trial have previously been found to be similar to those of Finnish patients with atrial fibrillation (Hallinen et al. 2014). Our study also broadens the available information regarding the impact of stroke severity on patients’ QoL and costs. To our knowledge, such data or results have not been published previously. Lastly, we presented the probabilistic results in terms of CEAF and EVPI.

There are always some limitations in cost–effectiveness assessments of chronic long-term illnesses that are based on modeling. AF is an especially complex disorder that has an impact on many health-related outcomes. Some aspects of the model used for this assessment can be considered conservative. An example of such an assumption is the modeling of most events as absorbing states. This assumption means that after experiencing an event the patients were no longer at risk of other costly events. Such an assumption tends to favor the treatment that has proven less effective.

There is also some uncertainty regarding the potential QoL loss and costs associated with warfarin monitoring. A recent Finnish study suggests that warfarin monitoring may result in some QoL loss (Soini et al. 2013), which was not accounted for in our analysis. Another recent study (Hallinen et al. 2014) shows that the Finnish national unit costs (Hujanen et al. 2008) used in this study for warfarin monitoring may not be generalizable across Finland and in fact may underestimate the true payer costs, at least for some municipalities. Since we excluded the possible QoL loss associated with warfarin, estimated warfarin treatment costs based on national unit costs, excluded potential benefits from the re-allocation of warfarin monitoring resources (i.e. the opportunity cost of labor needed in warfarin monitoring may be higher than its unit cost) for other purposes (Hallinen et al. 2012a, b), and the results were observed to be sensitive to warfarin treatment costs, our cost–effectiveness estimates may be conservative, at least for some municipalities.

## Conclusions

The ICERs obtained for apixaban were well below the WHO-CHOICE thresholds for cost–effective interventions, indicating that apixaban is a very cost–effective treatment alternative for warfarin in Finnish patients with AF. Based on the sensitivity, cost–effectiveness acceptability frontier, and expected value of perfect information analyses, the results were relatively robust; apixaban had a high probability of cost–effectiveness and the value of additional information for the probabilistic model parameters was relatively low.

## Methods

The cost–effectiveness assessment was performed using a life-time Markov health state transition model with 6-week cycles that extrapolate the trial findings to a longer term. Since the model structure has been previously described in detail by Dorian et al. (2014), it is summarized only briefly here. The assessment was based on the efficacy and safety findings from the ARISTOTLE trial (Granger et al. 2011) together with Finnish data for QoL, costs, and mortality. ARISTOTLE trial was a multicentre, randomized, double-blind trial, comparing apixaban (5 mg twice daily) with warfarin (target international normalized ratio, 2.0–3.0) in 18,201 patients with atrial fibrillation and at least one additional risk factor for stroke. Direct comparisons from randomized, double-blind trials are the favored data source for assessing health effects of compared treatments in Finland (Pharmaceuticals Pricing Board 2015), and ARISTOTLE trial is the only published phase III trial comparing aprixaban and warfarin in AF. We used secondary analysis of ARISTOTLE to derive model inputs (Dorian et al. 2014).

The main outcome measure was ICER calculated as the quotient of the mean cost and effectiveness differences between apixaban and warfarin. Since there are no official Finnish thresholds for cost–effectiveness (Soini et al. 2015) available, we applied the WHO-CHOICE thresholds in the assessment (WHO 2016). Accordingly, the intervention is considered very cost–effective, cost–effective or not cost–effective, when the ICER is below the gross domestic product (GDP) per capita (<37,576 euros/QALY gained based on the Finnish GDP in 2014 according to Official Statistics of Finland 2016), the ICER is 1–3 times the GDP per capita (37,576–112,728 euros/QALY gained) or the ICER is higher than three times the GDP per capita (>112,728 euros/QALY gained), respectively. The WHO-CHOICE thresholds are surprisingly similar to the overall results of the eight patient groups in a Finnish contingent valuation study, where also average WTP of 47,000 euros per QALY gained was observed for coronary heart disease (Soini et al. 2012). The effectiveness was estimated on the basis of QALY. QALYs were based on the survival and QoL was measured using EQ-5D-3L. All costs and outcomes were discounted at an annual rate of 3 %.

A probabilistic sensitivity analysis (PSA) with 2000 simulations was performed. In the PSA, the values of key input parameters were varied based on their probability distributions. A cost–effectiveness plane based on the PSA was drawn to illustrate the observed differences in costs and effects between compared treatments. In addition, a CEAF was drawn to depict the probability of cost–effectiveness for optimal treatments (in terms of net monetary benefit obtained) at different values of WTP per QALY gained (Barton et al. 2008; Soini et al. 2011). Conditional Bayesian Treatment Ranking (BTR) has been recently proposed as an assessment method for treatment ranking in terms of the probabilities for the first highest, second highest etc. benefits and the first lowest, second lowest etc. ICER versus minimum treatment (Soini et al. 2016). We used the BTR to define the probabilities for the first highest QALYs, lowest costs and lowest average cost–effectiveness ratios (CER). In addition, the EVPI was estimated to assess the value of obtaining more information (Barton et al. 2008) on probabilistic model parameters in order to reduce the uncertainty associated with the treatment decision. A high EVPI suggests that there is a need for additional information (i.e. the net monetary benefit gains from new research information may exceed the research costs) or that the opportunity cost should be considered in decision-making (Soini et al. 2011).

### Model

**Fig. 3:**
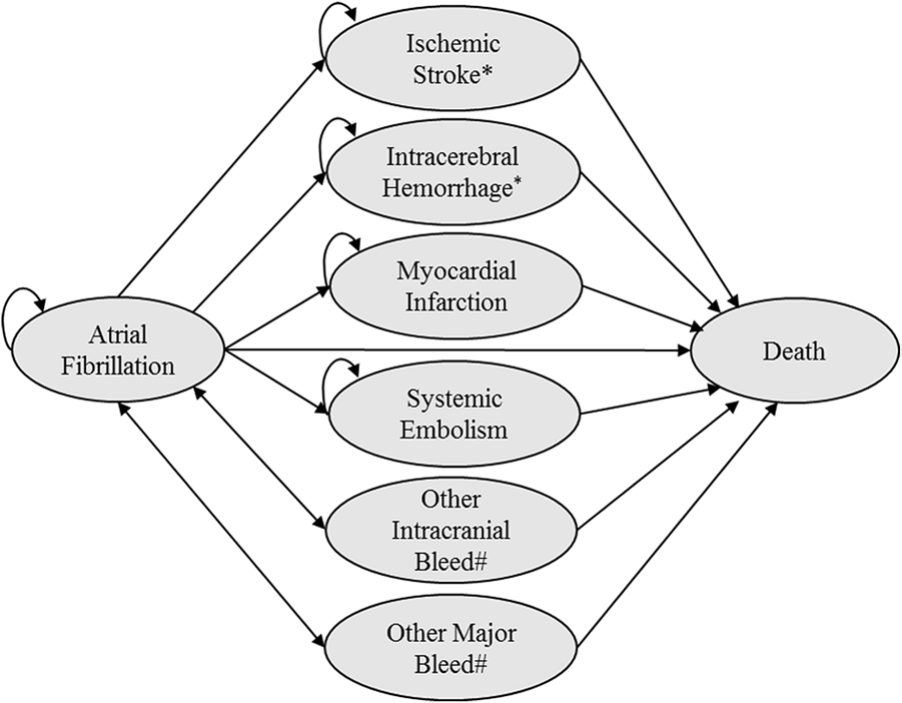
Schematic presentation of the cost-effectiveness model

A schematic presentation of the model used is presented in Fig. 3. The modeled cohort consists of male (64.7 %) and female (35.3 %) patients with non-valvular AF, aged 70 years, and with an average CHADS_2_-score (see Gage et al. 2001) of 2.1 (Dorian et al. 2014), which is well in line with the Finnish values (see Hallinen et al. 2014). In the model the patients start anticoagulation treatment and are modeled to reside in the “atrial fibrillation” health state until they die or experience one of the following permanent health events: ischemic stroke (mild, moderate, severe or fatal), hemorrhagic stroke (mild, moderate, severe or fatal), systemic embolism, or myocardial infarction (MI); or one of the following transient mutually exclusive events: other intracranial bleed (ICB), other major bleeds (gastrointestinal bleeds or other bleeds), or clinically relevant non-major bleeds. After permanent events the patients are no longer at risk of other events apart from death. After transient events the patients recover their previous health states after one cycle. The event rates are summarized in Table 2.

**Table 2:** Risk of modeled health events according to treatment

	ARISTOTLE Rate per 100 patient years^1^		Second line treatment Rate per 100 patient years	
	Apixaban	Warfarin	ASA^c^	No antithrombotic treatment
Ischemic stroke by CHADS_2_-score				
0–1 (34 % of patients)	0.521	0.458		
2 (35.8 % of patients)	0.950	0.934		
3–6 (30.2 % of patients)	1.534	1.944		
Weighted average/average	0.981	1.021	2.280	2.81^2^
Hemorrhagic stroke	0.254	0.512	0.388	–
Other intracranial bleed	0.076	0.288	1.455	–
Other major bleed: gastrointestinal	0.680	0.795	1.455	–
Other major bleed: not gastrointestinal	1.110	1.476	1.455	^–^
Clinically relevant non-major bleed	2.083	2.995	1.811	–
Myocardial infarction	0.530	0.610	0.616	0.856^3^
Systemic embolism	0.090	0.100	0.600	0.486^4^
Mortality for the trial duration^a^	3.0825	3.3404		
Other cardiovascular hospitalizations	–	–		
Treatment discontinuation^b^	13.177	14.405		

^a^Other cause mortality after the trial period was estimated by fitting a Gompertz survival function to the Finnish life tables

^b^For reasons other than modeled events

^c^The relative risk estimates from the AVERROES trial (Connolly et al. 2011) were applied to the apixaban event rates in the ARISTOTLE trial (Granger et al. 2011)

^1^Dorian et al. (2014)

^2^The ASA event rates were transformed using a relative risk (RR) reduction of 0.19 (Lip and Lim 2007) for ASA versus placebo

^3^The ASA event rates were transformed using RR = 0.72 (Yerman et al. 2007) for ASA versus placebo

^4^An RR of 0.19 for stroke was assumed to apply for SE as well

Mortality is modeled separately for the above health events (Dorian et al. 2014) and other causes. The other cause mortality is modeled on the basis of official Finnish life tables by applying a weighted relative risk of 1.24 for death due to AF as compared with the general Finnish population. The applied increase in mortality risk was based on the finding that AF increases the mortality risk by approximately 1.5-fold in women and 1.1-fold in men (Benjamin et al. 1998). Similarly, mortality is increased after stroke (both ischemic and hemorrhagic) and MI when compared with the general population. We derived the following standardized mortality ratios from the studies by Huybrechts et al. (2008) and Brønnum-Hansen et al. (2001): mild stroke 1.386, moderate stroke 2.440, severe stroke 6.384, MI women 2.330, and MI men 1.740. In addition, Finnish case fatality rates were applied for event rates that were not available from the ARISTOTLE trial. The case fatality rate for systemic embolism (10.5 %) was approximated from the Official Statistics of Finland (2010, 2011) and the case fatality rate for MIs was estimated from the Finnish National Cardiovascular Diseases Register based on year 2012 (14.2 % for men and 16.9 % for women). The Finnish 30 day-case fatality for MI among patients who make it to the hospital has been reported to be 13.5 % for men and 15.8 % for women (Häkkinen et al. 2007), but these rates exclude patients who die before being hospitalized.

In the model, the patients may discontinue their anticoagulation treatment due to modeled events or unrelated reasons after which they may switch to a second-line treatment [warfarin, acetylsalicylic acid (ASA)] or to no treatment. For simplicity we assumed that the patients would not receive further antithrombotic treatment after treatment discontinuance. Permanent treatment discontinuance was assumed for all hemorrhagic strokes whereas patients with other ICBs and other major bleeds were assumed to discontinue treatment either permanently or temporarily for 6 weeks as described by Dorian et al. (2014).

The model allows the adjustment of stroke and bleeding risks during warfarin treatment based on the TTR. The event rates for patients with different TTRs were obtained from a secondary analysis of the ARISTOTLE trial data, in which the trial centers were classified into four groups of equal size (i.e. 25 % of the study centers belonged to each group) based on the center’s TTR (cTTR < 52.38 %, 52.38 % ≤ cTTR < 66.02 %, 66.02 % ≤ cTTR < 76.51 %, and cTTR ≥ 76.51 %) (Dorian et al. 2014). By varying the proportion of patients belonging to each of these subgroups in the model, the impact of warfarin treatment quality on cost–effectiveness was assessed in a sensitivity analysis.

### Quality of life (QoL) estimates

QoL associated with health states was estimated from a nationally representative sample of Finnish inhabitants who participated in the Health 2000 study (Methodology report, Health 2000 Survey 2008).

In the model, the patients’ disability status after stroke is based on the modified Ranking Scale (mRS), which was not assessed in the Health 2000 study as such. Instead, we classified patients into the mRS categories on the basis of their answers to the ‘mobility’ and ‘usual activities’ dimensions of the 15D health-related QoL questionnaire (Sintonen 2001) as follows: answers 1–2 to both 15D dimensions was considered a mild stroke; answers 3–4 to either dimension was considered a moderate stroke, and answer 5 to either dimension was considered a severe stroke.

The QoL impact of the studied conditions in the Health 2000 study data was estimated using a regression method whereby the differences in utilities measured with EQ-5D-3L was explained with the conditions of interest. Due to difficulties in defining appropriate diagnoses for clinically relevant non-major bleeds and other major bleeds in the data set, the impact of these health events was not analyzed in the regression models. The QoL scores associated with health states are reported in Table 3. For probabilistic sensitivity analysis, the QoL values were modeled as beta distributions with large standard errors (20 % of the mean value).

**Table 3:** Applied average Finnish health state costs (year 2014 values) and quality of life values

Model state	Cost, €	EQ-5D-3L score (n = 5690)
Atrial fibrillation		0.743^a^
Monitoring visit (warfarin only)	38.39^b^	
Routine care, GP visit	116.82^c^	
Ischemic stroke		
Mild		−0.087
Acute Care, per episode	4429.23	
Long-term maintenance, per month	0	
Moderate		−0.198
Acute Care, per episode	7526.19	
Long-term maintenance, per month	943.31	
Severe		−0.644
Acute Care, per episode	7532.07	
Long-term maintenance, per month	4293.22	
Fatal	5338.33	
Haemorrhagic stroke		
Mild		−0.071
Acute Care, per episode	2628.91	
Long-term maintenance, per month	2429.02	
Moderate		−0.352
Acute Care, per episode	9218.05	
Long-term maintenance, per month	2128.67	
Severe		−0.578
Acute Care, per episode	9399.58	
Long-term maintenance, per month	3722.89	
Fatal	5564.97	
Systemic embolism		−0.084
Acute Care, per episode	2072.39	
Long-term maintenance, per month	104.08	
Other intracranial bleeds, per episode	4257.04	−0.168^d^
Other major bleeds	−0.168^e^	
GI bleeds, per episode	3448.80	
Non-GI bleeds, per episode	3448.80	
Clinically relevant non-major bleeds	2006.51	−0.0582^f^
Myocardial infarction		−0.005
Acute Care, per episode	5316.31	
Long-term maintenance, per month	525.00	

^a^In the regression model the constant term was 1.068, the disutility associated with AF was −0.045, and the decrease in QoL per year of age was −0.004. As a result, the QoL in AF state equals 0.743 (=1.068 − 0.004 × 70 − 0.045, where 70 is the average age of patients)

^b^Hallinen et al. (2006, 2012a, b)

^c^GP-visit at primary health care (Kapiainen et al. 2014)

^d^Disutility applied for 6 weeks

^e^Assumed to be equal to other intracranial bleeds. Disutility applied for 14 days

^f^Sullivan et al. (2011). Disutility applied for 2 days

### Costs

Health care payer perspective was used in the analysis. Direct health care costs excluding value added tax were included as costs in the analysis. The costs were assessed at real values in 2014 euros.

The applied daily drug cost was 2.77 euros for apixaban (5 mg twice daily) and 0.09 euros for warfarin (5 mg once daily). In addition, 1.67 and 1.80 % of the patients on apixaban and warfarin, respectively, experienced dyspepsia during treatment in the ARISTOTLE trial. Dyspepsia was assumed to require one general physician (GP) visit, to last for 3 months, and to be treated with pantoprazole 20 mg/day (19.54 euros/package for 3 month treatment). Routine follow-up of patients was assumed to consist of an annual GP visit (116.82€) and warfarin monitoring (38.39€) was assumed to take place 17 times per year (Hallinen et al. 2006). Similar monitoring frequencies have also been reported in other Finnish studies (Hallinen et al. 2014; Soini et al. 2013).

The cost estimates associated with health states (Table 3) were derived from the Finnish Hospital Discharge Register (see e.g. Sund 2012), which covers the public specialized health care in Finland, and the national hospital benchmarking database (Linna and Häkkinen 2008). First, all patients with a diagnosis of AF (WHO International Classification of Diseases 10 (ICD-10) code: I48, n = 30,680) were identified from the databases for 2007. Then the costs of care for the acute events, total health care costs for three consecutive years (1 year prior and 2 years following the event), and the proportion of patients who died within 1 year of the event were collected. Because the patients’ mRS were not available for cost estimation, we classified strokes as mild, moderate, or severe when the patient was discharged home, admitted to a rehabilitative institution or institutional care following the acute event, respectively. Since the health care costs for the first year are typically high due to acute event costs, use of first-year costs would overestimate the costs in successive years. Therefore the annual mortality adjusted maintenance costs (in 2007 values) following the events were estimated as follows: (1) the acute event cost were subtracted from the total costs for 2 years after the event to obtain 2-year maintenance costs; (2) annual maintenance costs were derived from 2-year maintenance costs by assuming that the costs during the first 2 years after events would be equal for patients who are alive at the start of each year [i.e. annual maintenance cost = 2-year costs/(1 + % of patients alive at the start of year 2)]; (3) the average health care costs for 1 year prior to the index event were deducted from the annual maintenance costs to obtain an estimate of the additional health care costs following the index events. The costs were then inflated into 2014 values with a factor of 1.1998 (Official Statistics of Finland 2015) and into monthly costs by dividing them by 12.

### Sensitivity analyses

In deterministic sensitivity analyses we tested the impact of the following changes to input variables: (a) varying CHADS_2_-scores, (b) varying TTR, (c) no treatment discontinuations after trial duration, and the cost of warfarin monitoring (d) increased and (e) decreased by 50 %.

## Abbreviations

AF: atrial fibrillation
ASA: acetylsalicylic acid
BTR: Bayesian treatment ranking
CEAF: cost–effectiveness acceptability frontier
GP: general physician
ICER: incremental cost–effectiveness ratio
QALY: quality-adjusted life year
EVPI: expected value of perfect information
ICB: intracranial bleed
INR: international normalized ratio
mRS: modified Ranking Scale
MI: myocardial infarction
PSA: probabilistic sensitivity analysis
QoL: quality of life
SE: systemic embolism
TTR: time spent in therapeutic range
WTP: willingness to pay
